# Effect of a novel commercial potassium-oxalate containing tooth-desensitizing mouthrinse on the microhardness of resin composite restorative materials with different monomer compositions

**DOI:** 10.4317/jced.52933

**Published:** 2016-12-01

**Authors:** Barış Karabulut, Deniz C. Can-Karabulut, Serkan Güleç, Cem M. Doğan

**Affiliations:** 1 Asst Prof., Gülhane Medical Academy, Haydarpaşa, Istanbul, Turkey; 2 Assoc Prof., Sancaktepe Dental Health Hospital, Sancaktepe Istanbul, Turkey; 3 Asst Prof., Department of Pediatric Dentistry, Çukurova University, Adana, Turkey; 4 Prof, Department of Pediatric Dentistry, Çukurova University, Adana, Turkey

## Abstract

**Background:**

The effects of mouthrinses on dental resin composites have been investigated extensively. However, there is little information available regarding the effects of ‘newly developed mouthrinse’ formulations on the microhardness of different monomer based composite systems. Therefore, the aim of this study was to investigate the effect of a novel potassium-oxalate containing desensitizing mouthrinse on the microhardness of different monomer based composite materials.

**Material and Methods:**

A hundred and twenty specimens (6mm in diameter and 2mm in height) were prepared for composite resin groups (methacrylate based, DX-511 monomer based and silorane monomer based) and for storage solution groups (artificial saliva and potassium oxalate-containing tooth-desensitizing mouthrinse). After allowing post-polimerization the baseline Knoop microhardness measurements for all specimens were recorded. The specimens were stored in 20 mL mouthwash and artificial saliva for 12 hours at 37ºC. The post-immersion microhardness values of all specimens were also recorded. Data were subjected to ANOVA/Scheffe’s test at a significance level of 0.05. The intra group (pre and post immersion values) comparison of the mean microhardness values of the specimens was done using Wilcoxon signed rank test.

**Results:**

The microhardness of the silorane based composite was not affected significantly (*p*>0.05). The hardness values of the DX-511 monomer based composite and the methacrylate based composite exhibited a slight but not significant microhardness change compared to the baseline values (*p*>0.05).

**Conclusions:**

Studies reported that the effect of mouthrinses on microhardness changes of composite resins may be material dependent, and the hardness change susceptibility of a restorative material may be attributed to its resin matrix or filler type. However, dental monomers as well as the oral care products have an ever-evolving technology and future studies should consider newer products. Potassium oxalate containing mouthrinses, especially alcohol-free ones, may be used safely with dental composites with newly developed low-shrink monomer compositions.

** Key words:**Microhardness, monomers, mouthrinses, potassium-oxalate.

## Introduction

The use of a chemical mouthwash may have a major effect on improving the oral health of the individual (1). However, the use of mouthrinses may produce deleterious side-effects on the surface and physical properties of restorative materials (2). According to Ozer *et al.* (3), the detrimental effects of mouthrinses on resin composites must be taken into account. Almeida *et al.* (4) reported that the degradation of resin composites involves water sorption that results in a decrease in hardness and this mechanism is mainly dependent on the composition of the ‘polymeric matrix’. They have mentioned that the formulation of commercially available mouthrinses contains various substances, such as water, antimicrobial agents, salts, preservatives and, in some cases, alcohol (2,4,5). ‘Alcohol’ in the mouthrinses and low pH may soften the resin composite restorative materials (5,6). According to Jyothi *et al.* (6) the ‘low pH’ of mouthrinses may act in the polymeric matrix of the resin composite, through catalysis of ester groups from dimethacrylate monomers present in the composition (Bis GMA, Bis EMA, UDMA and TEGDMA) and the hydrolysis of these ester groups may form alcohol and carboxylic acid molecules that may accelerate the degradation of the resin composite. ‘Water’ is also directly related to the composite organic matrix deterioration; the absorption of this liquid results in a widespread process within the composite ‘resin matrix’ that causes its degradation and results in lower physical and mechanical properties such as resin hardness (7). The formulation of the mouthwashes consists of water (7). It is also known that acid solutions may cause changes in the organic composition of resin composites (7).

The preservation of surface properties of the restorative material such as surface hardness as a mechanical property which is directly related to the wear resistance of the material mainly determines the ‘restoration durability’ (8). A decrease in the hardness of a material may result in premature failure of a restoration requiring its replacement (6).

The effects of mouthrinses on dental resin composites have been investigated extensively (2-5,6,9-11). However, there is little information available, to our knowledge, regarding the effects of ‘newly developed mouthrinse’ formulations on the microhardness of different monomer based composite systems. Therefore, the aim of this study was to investigate the effect of a novel potassium-oxalate containing mouthrinse on the microhardness of different monomer based composite materials.

Though new monomer technologies have been developed and some of them already introduced to the dental market, dimethacrylate-based composites still currently represent the vast majority of commercially available materials for direct restoration (12). For example, according to the researchers (13), the good mechanical properties observed for microhybrid type Filtek Z250 (3M/ESPE, St.Paul, MN, USA) are probably due to the high inorganic content in this composite, in combination with an organic phase composed of monomers with stiffer backbones, which are also capable of strong intermolecular interactions. However, the presence of TEGDMA as a diluent may contribute to the increased sorption because of the hydrophilicity of this monomer (13).

However, although resin composites have been progressively re-formulated to improve their mechanical and physical properties, shrinkage is still a disadvantage (14,15). Several low shrinking resin composites have been introduced (13,15). According to the manufacturers’ information, the filler content and polymerization shrinkage rate of these low-shrinkage composite resin restorative materials are different from those of the conventionally used composites (12,16).

Some of these materials still use Bis-GMA as the base monomer, but resort to greater filler loadings or absence of low molecular weight diluents to achieve lower shrinkage. The introduction of pre-polymerized resin filler is another attempt to reduce the shrinkage. Other approaches include the use of high molecular weight monomers, such as the methacrylate derivatives of dimer acid (13). Other alternative monomers have been proposed with epoxide ring-opening polymerization type chemistries (13) by replacing the chain-monomers in the composite matrix by ring-shaped molecules (17). One commercial example is a silora-ne-based composite, which polymerizes via a cationic mechanism virtually insensitive to oxygen inhibition and additionally having a siloxane core to which the oxirane rings are attached making the molecule fairly hydrophobic (13). Oxirane groups which are stable in biological fluids probably due to their lack of solubility also decrease composite solubility and water absorption showing lower values than that presented by the dimethacrylate-based composites (17,18). The ring-opening polymerization of this composite instead of free radical polymerization of methacrylate monomers reveals low polymerization shrinkage (19). The fillers in Filtek Silorane (3M/ESPE, St.Paul, MN, USA), consist of 0.1-2.0 μm quartz particles and radiopaque yttrium fluoride. It contains 55% volume (76% weight) inorganic fillers with a particle size between 0.1 and 2 μm. Furthermore, changes throughout the inorganic phase may decrease the material’s physical properties, such as microhardness (7). One high molecular weight monomer called DX-511, based on urethane dimethacrylate and present in the commercial composite Kalore (GC Co, Tokyo-Japan), leads to shrinkage reduction due the low reactive group concentration available for reaction and has been shown to lower polymerization stress and shrinkage values compared with conventional composites (18). Also a reported upgrade in the mouthrinse formulation with fluoride, essential oils and xylitol will further enhance the treatment effect (20). The investigated null hypothesis was that microhardness values of different monomer systems would be affected with the immersion in a desensitizing mouthrinse.

## Material and Methods

Three commercial photo-activated resin composites with shade A2 were selected on the basis of their matrix monomer compositions.  Table 1 shows this different monomer based composite restoratives that were selected for this study. Filtek Silorane (3M/ESPE, St.Paul, MN, USA) was based on silorane, Kalore (GC Co, Tokyo-Japan) was based on a higher moleculer weight monomer DX-511 (UDMA based) and Filtek Z250 (3M ESPE, Dental Products, St.Paul, MN, USA) was based on aromatic and aliphatic dimethacrylates.  Table 2 shows the components of an alcohol-free essential oil/phenolic compound mouthrinse (Johnson & Johnson, UK) and artificial saliva selected as the immersion solutions. Forty cylindrical specimens for each composite material group; a total of 120 cylindrical specimens were fabricated 6 mm in diameter and 2 mm in thickness using a teflon mold as required by the ISO International Standard #7491:2000.17 Composite materials were left to stand for a few minutes at normal room temperature. Resin composite materials were applied carefully into a circumferential teflon mold with the same specimen dimensions positioned onto a 0.05 mm-thick transparent polyester filmstrip (Mylar, DuPont, and Wilmington, DE, USA) over a glass slide. Materials were covered with another celluloid strip, and the glass slide weighed of 200 g for 1 minute until the slide touched the mold completely, thus allowing excess composite to flow prior to curing (21) and to minimize the oxygen inhibition and maximize the surface smoothness (16). The distance between the light and the specimen was also standardized by using this 1-mm glass slide (22).

Next, the excess restorative material was removed. The restorative materials were light cured for 40 sec with a quartztungsten-halogen (QTH) light curing unit (HiluxUltra Plus, Benlioglu Dental, Istanbul, Turkey) in standard mode on each side with a conventional type tip. According to its manufacturer Filtek Silorane should not be cured with a plasma arc light or laser. The light intensity of the unit was monitored with a radiometer (Curing Radiometer, Model 100, Demetron/KerrCorp. Danbury, CT, USA) throughout the experiment and did not drop below 550mW/cm2 (energy density = 22 J/cm2). The radiant exposure was calculated as the product of the irradiance of the curing unit by using a radiometer and the time of irradiation. Immediately after polymerization, the specimens were stored in distilled water in a dark container that was maintained in a humidor at 37°C for 24 hours, allowing post-polymerization, prior to the baseline microhardness measurement. After 24 hours, the specimens were subjected to surface polishing with abrasive disks (Sof-Lex, 3M ESPE, St. Paul, USA) with continuous water irrigation in decreasing order of abrasiveness (10 s each), in a slow-speed handpiece (23). Polishing procedures were kept to a minimum time, 10 s for each step, to avoid micro crack formation (23). The samples were inspected visually before and after testing to confirm the absence of any surface defects or pores. After that the baseline values were taken for each disc. The initial microharness measurements for each specimen prior to immersion in any treatment solution were performed and recorded as baseline measurements and compared to the test values at the end of 12 hours.

**Table 1:**
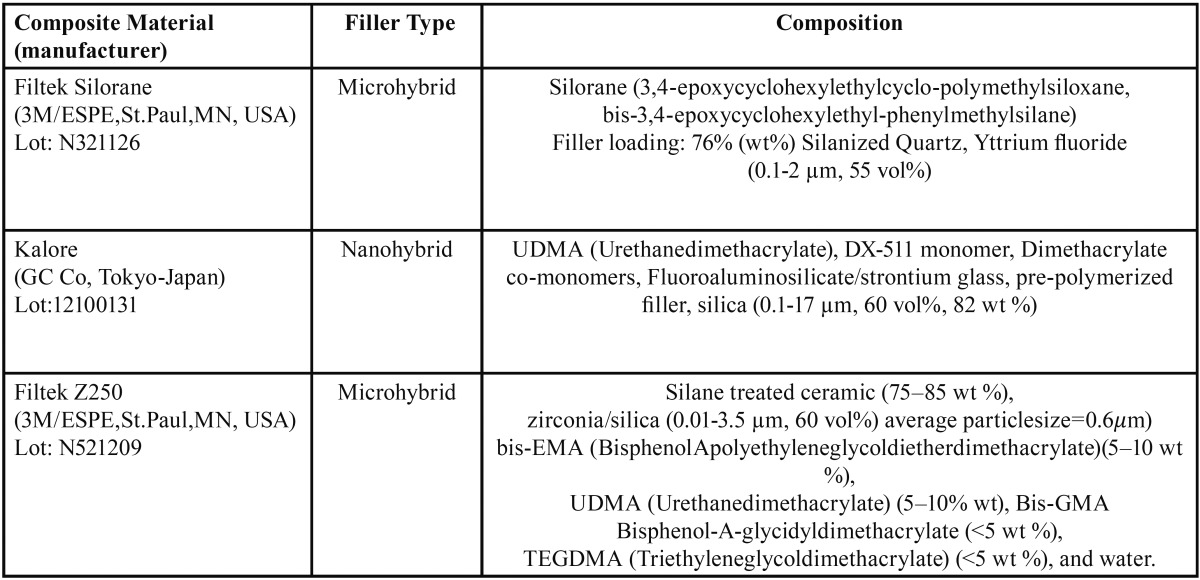
Composition of the resin composites tested.

**Table 2:**
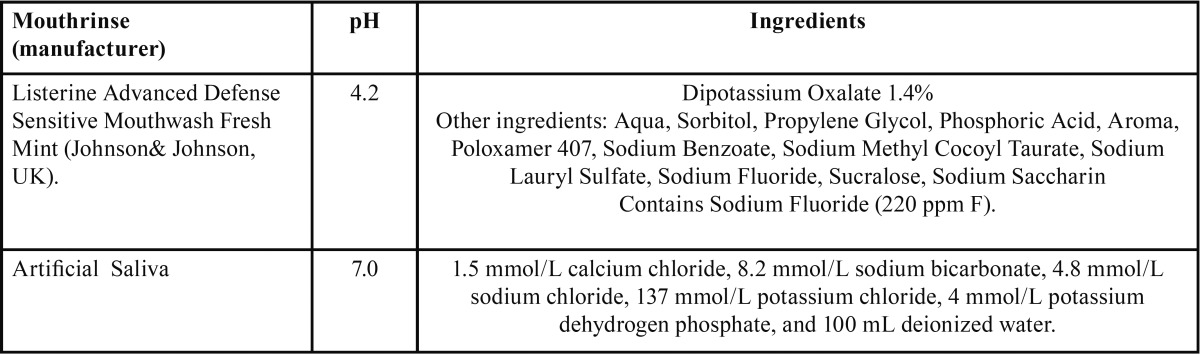
Composition and pH of immersion mediums used in the study.

Following the baseline measurements, prepared specimens were randomly divided into three groups according to the type of direct resin-based restorative material used and were randomly subdivided into two different subgroups (n = 20) in terms of immersion medium (20 mL). Immersed specimes were kept in a dark glass container that was maintained in an incubator at 37°C for 12 hours, which is equivalent to a cumulative time period of 1 years of 2-minute daily use of mouthrinse (21,24). Test solutions were shaken every 3 hours to provide homogeneity (22). The artificial saliva contained an electrolyte composition similar to that of human saliva and a pH of 7.0. (25). After this immersion period twenty milliliters of distilled water was used to thoroughly rinse each specimen for 120 seconds (21). Each specimen was then blotted dry using a filter paper, and then subjected to microhardness test. Initial Knoop microhardness number (kg/mm2) readings were obtained (5,10,26,27). Knoop hardness was measured at three different locations on each specimen and the mean Knoop hardness determined from three readings (5) (Buehler Mmt-3, Waukagen Lake, Bluff, Il, USA). A 50 gf load was applied with a dwell time of 15 s (26). Post-immersion Knoop hardness readings were obtained from each specimen using the same method which was used in the initial Knoop hardness readings. After three readings, the microhardness mean values of the test specimens were obtained. All microhardness measurements were performed by the same operator, and the mean values were used for the subsequent statistical analysis. For multiple comparisons, means and standard deviations were calculated. Two-way analysis of variance (ANOVA) was used to determine the effect of the interaction between materials and medium on microhardness with SPSS (version 15.0.1; SPSS, Chicago, IL, USA). One-way ANOVA and post hoc Scheffe’s test were used to determine the inter-medium differences at a significance level of 0.05. The intra group (pre and post immersion values) comparison of the mean value of microhardness of the specimens was done using Wilcoxon signed rank test. A *p* value of < 0.05 was considered statistically significant. Means, standard deviations, and significant differences in microhardness are presented in  Table 3.

**Table 3:**
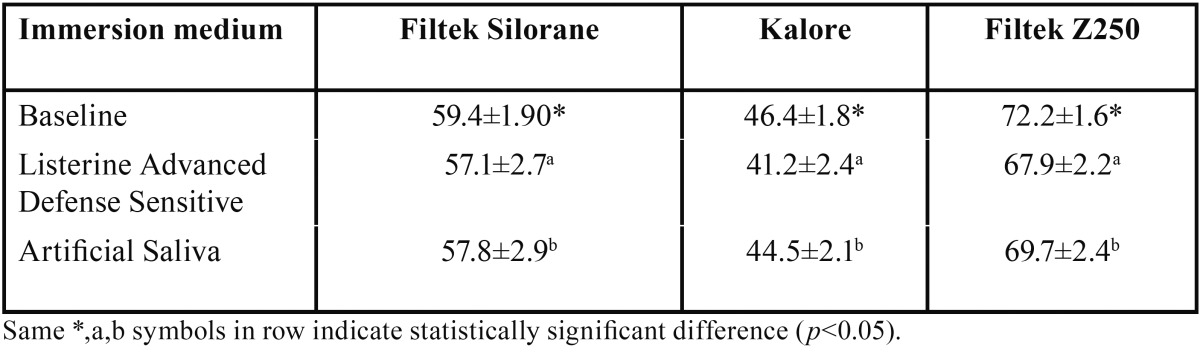
Intra (column) and inter (row) group comparison of means, standard deviations, and significant differences in microhardness in two different immersion mediums (KHN-Kgf/mm2).

## Results

 Table 3 shows the mean Knoop microhardness readings of dental composites after immersion in the mouthrinse and artificial saliva. No significant reduction in the microhardness was observed in all the groups after immersion in the mouthrinse compared to baseline values with *p*> 0.05. There were no significant interactions between the test solutions and the test materials with regard to microhardness (*p*> 0.05) The microhardness of composite materials was affected by the mouthrinse however the differcence was not significant (*p*>0.05). On the other hand, the baseline microhardness values among composite systems were significantly different; Filtek Z250 demostrated the highest baseline microhardness value which was followed by Filtek Silorane and Kalore (*p*<0.05). Post immersion values among groups were similar to the baseline values following the same order (*p*<0.05). The mouthrinse found to have no statistically significant effect on any of the materials used. There was a slight difference between the articial saliva and mouthrinse values however this difference was not found to be statistically significant.

## Discussion

Taking into account the results of this study, the investigated null hypothesis, suggesting that the microhardness values of composites with different monomer systems would be affected with the immersion in a desensitizing mouthrinse could not be accepted.

No significant reduction in the microhardness was observed in all the groups after immersion in the mouthrinse compared to baseline values with *p*> 0.05. This may be because of the ‘alcohol-free’ structure of the mouthrinse studied. More recently, for various reasons there has been an increase in the demand for alcohol-free mouthrinses (1). Potential problems with alcohol rinses that are being reported include the softening of the tooth-colored restorations (1,4,7). Ethanol, which is found in many mouthrinses, may accelerate the hydrolytic degradation of resin-based materials (3). Another factor may be the potassium-oxalate content of the studied mouthrinse. Subsequent work showed that the presence of soluble potassium-oxalate led to the formation of insoluble calcium oxalate precipitates having the added advantage of being relatively insoluble in acid (28).

There was a slight difference between the articial saliva and mouthrinse values for Filtek Z250 and Kalore although this difference was not found to be statistically significant. This slight difference may be attributed to the lower pH of the mouthrinse (pH:4.2) compared to the artificial saliva (pH:7.0). ‘The low pH’ of essential oil mouthrinses may have the potential for erosion (1,6). This phenomenon is a complex process that might result in composite polymer matrix collapse, causing several problems such as filler-polymer matrix debonding, release of residual monomers, and wear and erosion (21). The higher acidity may alter the polymeric matrixes of the resin composite by catalysis of ester groups from ‘dimethacrylate monomers’ present in their compositions. The hydrolysis of these ester groups may form alcohol and carboxylic acid molecules, which accelerate the degradation of the resin composites, due to the decrease of pH inside the resin matrix (7). In addition to pH, mouthrinses can contain ‘other substances’, such as detergents, emulsifiers, and organic acids, which can lead to the degradation of the composite resin surface (21). Researchers (5) also reported that despite the absence of alcohol, ‘phosphoric acid’ in the mouthrinse composition may alter the polymer matrix of composites by catalysis of the ester groups present in the ‘dimethacrylate monomers’. The degradation of the polymer network leads to a phenomenon called plasticization which decreases microhardness values in composites (5). The mouthrinse used in this study has also phosphoric acid in its composition and this might be the reason for the reported slight difference. The adverse effect of phosphoric acid on the filler- resin interface may result in filler matrix debonding by water uptake. Subsequently, displacement of the filler particles can occur. This phenomenon may lead to a decreased microhardness. The formulation of the mouthwashes also consists of water (7). The monomer type also directly influences the potential water sorption of the material. Monomers like UDMA, BisGMA, and TEGDMA contain polar groups such as -OH-, -O-, and -NH-. These groups increase the material’s hydrophilicity, probably making it more prone to salivary sorption (2). However, the results were not statistically significant in our study and this may be because of lower water sorption values reported for Filtek Z250 in the literature. The higher filler content and thus lower organic content, and the greater hydrophobicity of the organic phase, by the use of mostly BisEMA in place of BisGMA, may have been the reason of lower sorption values (3,13). In addition, the smaller the filler particle, the smaller the amount of water absorbed by the polymer network, which results in lower degradation of the interface matrix/particle. The Z250 contains filler particles with average size of 0.6 micrometer (5). Larger fillers might be easily eroded by the chemical actions of the mouthrinses, leading to rougher surfaces (21). The filler type such as particles of zirconia instead of barium glass as filler may also have influenced the Knoop hardness values (13). The filler particles and the resin matrix of a composite and the characteristics of these particles have a direct impact on surface hardness (24).

Filtek LS on the other hand, was also reported to have lower sorption values, more likely due to the presence of more hydrophobic monomers (13), and resistance to ethanol degradation may also be attributed to low water sorption, which is a result of the absence of more hydrophilic monomers, such as TEGDMA and UDMA. Also the high filler loading might have minimized the sorption of solvent, thus leading to smaller reductions in the mechanical properties (13).

However, Son *et al.* (29) reported that Filtek Silorane had the lowest microhardness, despite having the highest degree of conversion among the specimens examined because it has the lowest filler content. Similar to our study, they found that Filtek Silorane showed lower microhardness values than Filtek Z250 and mentioned that the difference in the microhardness values between silorane and other tested methacrylate based composite could be attributed to the filler content. However, there is a controversy regarding the surface hardness of the Silorane composite (8). According to Shafiei *et al.* (8) although the Silorane composite contains 5% lower filler than that of the methacrylate composite; the highly cross-linked polymer matrix originating from the multifunctional Silorane monomer and its hydrolytic stability may account for the comparable hardness values obtained. In the current study, microhardness of Silorane composite was not altered after mouthrinse immersion. This result may be explained based on the high chemical stability and hydrophobicity of Silorane matrix.

The type, chemistry, morphology, and size of the fillers have been reported to affect the material’s surface hardness (8). The low surface hardness value of the Kalore composite may be attributed to the high resin monomer content. The molecular weight of the monomer (DX-511) in Kalore was increased in order to reduce polymerization shrinkage however DX-511 monomer is a chemically changed UDMA monomer. Water absorption of Kalore may be similar to UDMA based composite resins. Moreover, Sun *et al.* (20) also reported that it is important that the composite resin presents uniform filler particle distribution in the polymer network to minimize the formation of filler-rich and filler-depleted areas within the composites. This is especially important regarding the performance of composites in aqueous solutions, since voids or nonbonding spaces at the filler/matrix interface may increase the water sorption of composites (5). Kalore has an un-uniform dispersion of fillers consisting of pre-polymerized fillers and different size fillers. Takahashi *et al.* (30) compared the basic mechanical characteristics of nanofiller prepolymer containing composite Kalore with microhybrid Filtek Z250 and reported that the mechanical performance of the microhybrid material Z250 was overall slightly better than the nanohybrid material.

Bis-Gma free low shrinking resin composite Kalore is based on a novel monomer (DX-511), which is a modified UDMA and has a high molecular mass in comparison to Bis-GMA (895 g/mole vs. 512 g/mole) (15). According to the manufacturer the modified strontium glass reinforces the filler’s strength and surface hardness. The molecular weight of DX-511 (Mw 895) is twice the molecular weight of bisGMA or UDMA, reducing polymerization shrinkage since a smaller number of carbon double bonds (C=C) are present. DX-511, the low shrinkage monomer, is effective in reducing shrinkage stress. The reduction in ongoing stress within the composite resin helps retain fillers in the matrix, especially after stress is applied to the cured composite resin. That is why the surface smoothness and wear resistance were found to be superior with the addition of DX-511 to the composite resin formulation.

It is difficult to compare the results of this study with data from the literature, as there are no published studies available where these four systems (three composites and one newly developed mouthrinse) were compared with one another. The effect of mouthwashes on knoop hardness changes of composite resins may be material dependent, and the hardness change susceptibility of a restorative material may be attributed to its resin matrix or filler type. However, within the limitation of this laboratory study; based on the employed methodology and the obtained results the findings of the reported research disclosed that a new potassium- oxalate containing mouthrinse did not altered, to some degree, the microhardness of the tested resin composites. The use of an alcohol-free mouthrinse may be preferable for patients with extensive composite restorations (3). However, results for the same material may differ greatly between methods due to differences in testing configuration and instrument compliance (15). When discussing the clinical relevance of these results, the oral environment must be considered, as it differs in several ways from in vitro conditions (21). Factors such as the variety of food, saliva, and their interactions may change the results (21). Future studies should consider longer periods of immersion (22). Better standardization and reporting of newly developed mouthrinses with randomised clinical trials are necessary in longer-term follow-ups.
